# Femoral anteversion linked to the inability to squat: Analysis of CT images in the patient and control groups

**DOI:** 10.1097/MD.0000000000038411

**Published:** 2024-06-07

**Authors:** Dong Hwan Lee, Yun Hwan Kim, Jaeyoon Baek, Seon Ae Kim, Seok Jung Kim

**Affiliations:** a Department of Orthopedic Surgery, Yeouido St. Mary’s Hospital, College of Medicine, The Catholic University of Korea, Seoul, Republic of Korea; b Department of Orthopaedic Surgery, Uijeongbu St. Mary’s Hospital, College of Medicine, The Catholic University of Korea, Cheonbo-ro, Uijeongbu-si, Gyeonggi-do, Republic of Korea.

**Keywords:** excessive out-toeing squat, femoral torsion, femur retroversion, squat, tibial torsion

## Abstract

Many patients who cannot squat well in a neutral toe position can only squat in an excessively out-toeing position. This excessive out-toeing squat is thought to be caused by rotational problems of the lower extremities. In this study, we aimed to identify the cause for the inability to squat by measuring and comparing femoral and tibial torsion between an excessive out-toeing squat patient group and a control group representing the general population. Between 2008 and 2022, a patient group comprising 50 lower extremities with excessive out-toeing squats was established. A control group representing the general population was selected from patients aged 0 to 29 years, who underwent lower-extremity CT angiography between 2012 and 2022, using the Clinical Data Warehouse with exclusion criteria applied. A total of 94 lower extremities were included in the control group. The femoral torsional angle (FTA) and tibial torsional angle (TTA) of both groups were measured and compared using Student *t* test. Additionally, 30 each of those with the highest and lowest 30 FTA values were selected from the patient and control groups, and the TTA was compared between the high- and low-FTA groups using Student *t* test. The mean FTA was 0.34° (SD, 11.11°) in the patient group and 10.14° (SD, 11.85°) in the control group, with a mean difference of 9.8° and *P* < .001. The mean TTA was 27.95° (SD, 7.82°) in the patient group and 32.67 ° (SD, 7.58°) in the control group, with a mean difference of 4.72° (*P* = .001). The mean TTA was 34.3° (SD, 7.72°) in the high-FTA group and 28.17° (SD, 8.35°) in the low-FTA group, with a mean difference of 6.13° (*P* = .005). Patients with excessive out-toeing squat showed lower FTA and TTA values than the general population. Furthermore, although a correlation between FTA and TTA was not established through Pearson correlation analysis, a tendency was observed where a decrease in FTA was associated with a decrease in TTA. Based on these results, decreased FTA was demonstrated to be one of the major causes of excessive out-toeing squats.

## 1. Introduction

### 1.1. Background

**Figure 1.**
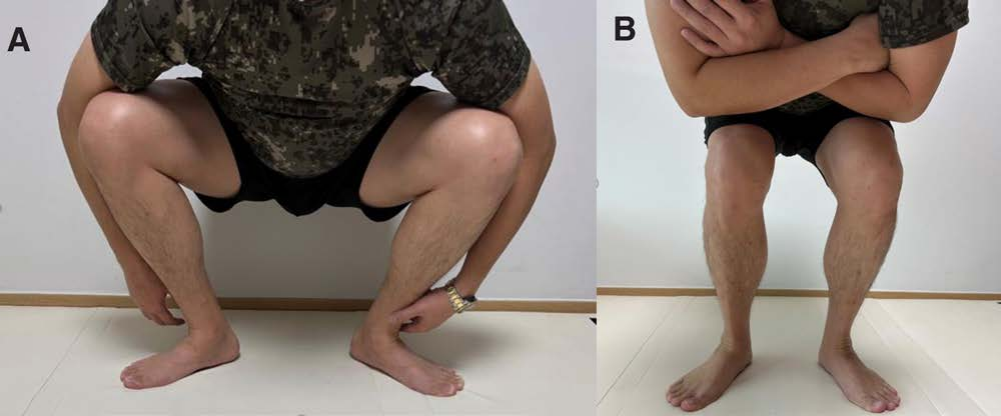
Clinical photograph showing the squatting posture of a patient from the patient group. (A) Squatting in an excessive out-toeing position. The hip is observed to descend below the knee, allowing for a full squat. (B) Squatting in a toe-neutral position. The hip is observed to position above the knee, indicating an inability to squat completely.

Many children and young adults who visit outpatient clinics complain of difficulty in squatting. They often have reduced athletic ability or experience hip pain during exercise because of their limited squatting position. Upon physical examination, decreased femoral torsion is suspected in most cases. Furthermore, when these patients were asked to squat, they often assumed an excessively out-toe position and squatted with their hips in an externally rotated state (Fig. 1). Patients who experience hip pain due to the inability to squat face a significant challenge, especially in real-world scenarios such as military service. Many countries around the world still maintain conscription systems.^[1–5]^ It is a well-known fact that military training involves intense physical demands. Consequently, individuals experiencing an inability to squat or encountering repeated hip pain during squatting may face considerable discomfort upon enlistment.

### 1.2. Rationale

Although there is no conclusive research on the cause of excessive out-toeing squats, studies have identified gluteus muscle contracture (GMC) as one of the possible causes.^[6,7]^ Chiang et al^[2]^ suggested aberrant femoral torsion as another probable cause of excessive out-toeing squat. In their study, diminished femoral anteversion and femoral retroversion were determined based on the normal values established in previous studies,^[8–10]^ and aberrant femoral torsion was observed in 30% of the study participants. However, no comparisons were made to the general population. Because the FTA is known to have a large variance,^[8,11]^ various factors such as racial differences are likely to affect the average value in the general population.^[12]^ Therefore, we aimed to investigate the differences in the FTA between patients with excessive out-toeing squats and the general population.

Internal hip rotation and decreased tibial torsion are known as the leading causes of in-toeing gait in children.^[13–15]^ Similar to the in-toeing gait, rotational problems of the lower extremities can also contribute to excessive out-toeing squatting. Therefore, we aimed to investigate whether the general squatting position was also limited in cases of decreased tibial torsion and whether excessive out-toeing squatting was likely to occur. Additionally, we hypothesized that with decreased FTA, tibial torsional angle (TTA) would also be reduced, and we aimed to investigate this relationship.

### 1.3. Objectives

Therefore, we made 3 assumptions: the FTA of the excessive out-toeing squat group would be lower than that of the general population; the TTA of the excessive out-toeing squat group would be lower than that of the general population; and if the FTA decreased, the TTA would also decrease. This study aimed to prove these assumptions and elucidate the relationship between excessive out-toeing squats, FTA, and TTA. Based on this, we aimed to highlight decreased femoral torsion as a novel cause of the inability to squat.

## 2. Materials and methods

### 2.1. Study design and setting

The reporting of this study conforms to the STROBE statement. This was a retrospective study that compared patients squatting in excessively out-toeing positions with controls representing the general population. Data for the patient group were extracted from the database of Uijeongbu St. Mary’s Hospital, and data for the control group were extracted from the Clinical Data Warehouse (CDW) of the Catholic Medical Center Information System (CMC nU).

### 2.2. Participants

#### 2.2.1. Patient group

We examined the rotation profiles and Achilles tightness in patients who visited our outpatient clinic with concerns of difficulty in squatting between May 2008 and May 2020. Patients with Achilles tightness or other measurement limitations due to trauma were excluded from the study. To examine the rotation profiles, we measured the angle of hip internal rotation with the knee flexed to 90° in the prone position (Craig test). Patients who exhibited an excessive out-toeing squat and showed even a slight limitation of internal rotation compared to normal individuals on physical examination were classified as the patient group, and a CT scan was performed for the torsional study. The squat assessment was conducted as follows: subjects were instructed to squat in a foot neutral position and were selected if the hip did not descend to knee level or lower; subjects were asked to adopt an out-toeing position greater than the neutral position, and were selected if it was possible for the hip to descend to the knee level or below during the squat. Patients who met both of these criteria were considered to exhibit an excessive out-toeing squat. Women were excluded from the analysis owing to the small sample size of only 2 female patients and expected sex-based anatomical differences. The patient criteria are outlined as follows. The inclusion criteria are: male; patients exhibiting an excessive out-toeing squat during the squat assessment; patients with any degree of limitation in hip internal rotation as assessed during the physical examination. The exclusion criteria are as follows: patients with Achilles tightness; patients whose evaluation of torsional abnormality is restricted due to trauma, surgery, etc. The CT protocol is as follows: CT scans were performed for those included in the patient criteria, with axial images taken bilaterally at the hip, knee, and ankle joint levels. Finally, we analyzed 50 femurs from 25 patients who underwent CT because of suspected torsional abnormalities based on physical examinations.

#### 2.2.2. Control group

The CDW of the CMC nU was used to extract data for FTA and TTA measurements in the general population. The following inclusion criteria were used: patients who visited the relevant hospitals between October 2012 and October 2022; male patients only; and patients aged 0 to 29 years who underwent lower-extremity CT angiography. CT data were extracted from patients who met both criteria. A total of 74 CT datasets were obtained, and all the data were anonymized before being provided. No other criteria were set, and patients with fractures or implants in either leg or those in whom the torsional angle could not be measured from the CT image were excluded. Finally, a control group of 47 patients and 94 lower extremities was established for FTA measurement and analysis.

### 2.3. Experiment description

#### 2.3.1. Radiologic measurements

All patients underwent CT in the supine position, and the PACS software (ZeTTA PACS, TaeYoung Soft Co., Anyang-si, Gyeonggi-do, Korea) was used for angle measurements. The angles of the control group were measured using the Online DICOM viewer (I-Rapha, IRM Inc., Seoul, Korea) as the data were obtained from the CDW.

#### 2.3.2. Femoral torsional angle

**Figure 2.**
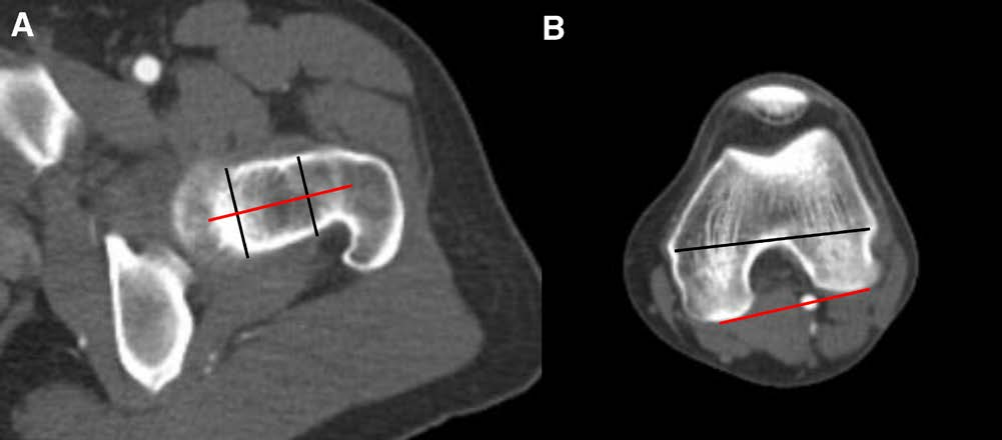
Method for measuring the femoral torsional angle. (A) On the axial image where the femoral neck appears longest, a line is drawn connecting the head center and neck center (red line). (B) On the axial image where the trans-epiphyseal axis of the distal femur is most clearly visible, a posterior condylar axis is drawn by connecting the most posterior points of both condyles (red line). The angle formed by the 2 red lines in (A) and (B) is measured to obtain the final femoral torsional angle. The femoral torsional angle in this figure is decreased, showing femoral retroversion.

The femoral neck axis was defined as the line connecting the neck center on the trochanteric side and the neck center on the head side in the CT axial image, where the femoral neck was the longest. The posterior condylar axis was defined as the line connecting the posterior borders of both femoral condyles in the CT axial image, where the distal femoral transepicondylar axis was most clearly visible. The angle between the 2 axes was measured to determine the FTA (Fig. 2).^[16]^

#### 2.3.3. Tibial torsional angle

**Figure 3.**
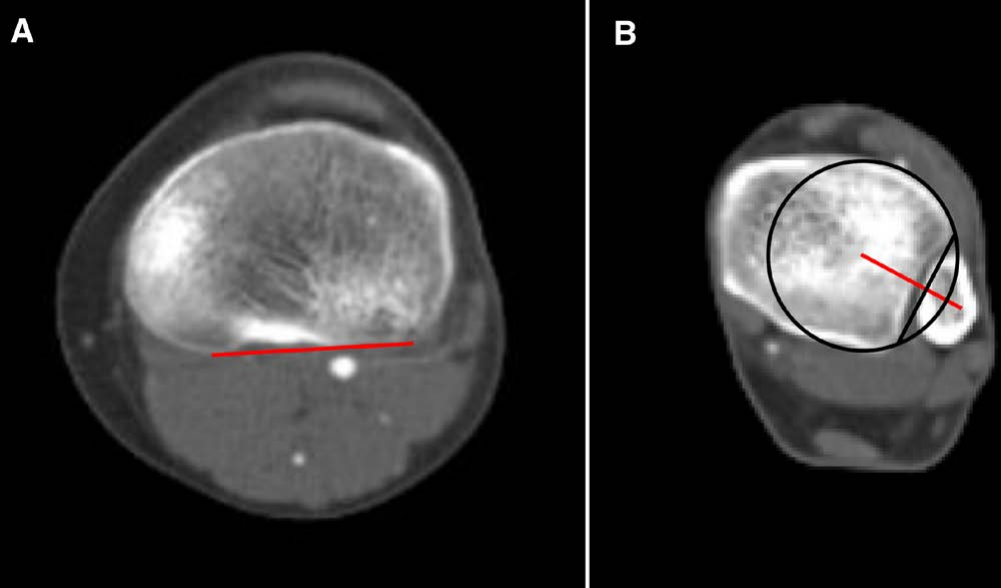
Method for measuring the tibial torsional angle. (A) In the axial image of the proximal tibia just above the head of the fibula, a line is drawn connecting the most posterior points of both condyles (red line). (B) In the axial image of the distal tibia just above the talocrural joint, a line is drawn connecting the center of the tibial pilon circle and the center of the fibular notch (red line). The angle formed by the 2 red lines in (A) and (B) is measured to obtain the final tibial torsional angle.

To prove the second and third assumptions, the TTA of the patient and control groups were measured and compared. Additionally, to investigate the relationship between FTA and TTA, 30 each of those with the highest and lowest FTA values were selected from the patient and control groups, and the TTA was measured and compared between the high- and low-FTA groups. Jend CT method was used to measure TTA.^[17,18]^ First, the axis connecting the posterior border of the proximal tibia was determined on the axial CT image immediately above the fibular head. The axis connecting the center point of the tibial plafond, excluding the medial malleolus, and the midpoint of the fibular notch was determined in the CT axial image just above the talocrural joint space. The angle between the 2 axes was measured and defined as the TTA (Fig. 3).

### 2.4. Statistical analysis

FTA and TTA measurements were performed by 2 orthopedic surgeons with sufficient knowledge of the measurements. Two observers independently performed the measurements without sharing their values, and 1 observer repeated the process to obtain 3 sets of measurements. Three sets of measurements were averaged for the final analysis. SPSS for Windows (SPSS version 26, SPSS Inc., Chicago, IL, USA) was used for statistical analysis. First, the Student *t* test was performed to evaluate whether the FTA and TTA values in the patient group were significantly lower than those in the control group, which represents the general population. The analysis was conducted using the Student *t* test because it simply compares the magnitude of measurements between 2 groups. Second, Pearson correlation analysis was performed to evaluate the correlation between the FTA and TTA. Third, the Student *t* test was performed to evaluate whether the TTA values were significantly different between the high-FTA and low-FTA groups. To examine the association between FTA and TTA, a Pearson correlation analysis was conducted. Furthermore, from the pooled data of both groups, 2 new groups were formed: 1 consisting of the 30 subjects with the highest FTA measurements and the other consisting of the 30 subjects with the lowest FTA measurements. The TTA values of these 2 newly established groups were then compared. Since this comparison simply involved comparing the magnitude of measurements between the 2 groups, the Student *t* test was utilized for the analysis.

### 2.5. Demographics

The patient group had 25 men with a mean age of 17.4 years (7–31 years), and the control group consisted of 47 men with a mean age of 21.89 years (8–29 years).

## 3. Results

### 3.1. Interobserver and intraobserver reproducibility

The ICC was calculated to assess reproducibility and reliability. For the FTA, the ICC for interobserver variability was 0.984 and that for intraobserver variability was 0.980. For the TTA, the ICC for interobserver variability was 0.993 and that for intraobserver variability was 0.990 (Table 1).

**Table 1:** Reliability of femoral torsional angle and tibial torsional angle measurements.

	Interobserver			Intraobserver		
	ICC	95% CI	*P* value	ICC	95% CI	*P* value
Femoral torsional angle	0.984	0.974 to 0.990	<.001	0.980	0.966 to 0.988	<.001
Tibial torsional angle	0.993	0.989 to 0.996	<.001	0.990	0.983 to 0.994	<.001

### 3.2. Comparison between the patient and control groups

First, the patient and control groups were compared. The mean FTA was 0.34° (SD, 11.11°) in the patient group and 10.14° (SD, 11.85°) in the control group, with a mean difference of 9.8° and *P* < .001, indicating a statistically significant difference between the 2 groups. The mean TTA was 27.95° (SD, 7.82°) in the patient group and 32.67° (SD, 7.58°) in the control group, with a mean difference of 4.72° (*P* = .001), indicating a statistically significant difference between the 2 groups (Table 2).

**Table 2:** Comparison of femoral torsional angle (FTA) and tibial torsional angle (TTA) in the patient and control groups: results from Student *t* test.

	Patients group (mean ± SD)	Control group (mean ± SD)	*P* value
Femoral torsional angle	0.34° ± 11.11°	10.14° ± 11.85°	<.001
Tibial torsional angle	27.95° ± 7.82°	32.67° ± 7.58°	.001

### 3.3. Analysis of the correlation between FTA and TTA

Next, the correlation between FTA and TTA was analyzed. First, Pearson correlation analysis between FTA and TTA yielded a coefficient of correlation of 0.197, with *P* = .019, indicating no significant correlation. This can be attributed to the limited range of FTA and TTA distribution, making them susceptible to even small errors, as well as the overall small sample size. Therefore, 2 new groups were created by pooling the entire dataset, each consisting of 30 patients with high FTA and low FTA. Student *t* test was performed to compare the differences in TTA between these 2 groups to further investigate the relationship.

The mean FTA was 20.62° (SD, 5.46°) in the high-FTA group and − 12.74° (SD, 5.79°) in the low-FTA group, with a mean difference of 33.36°. The mean TTA was 34.3° (SD, 7.72°) in the high-FTA group and 28.17° (SD, 8.35°) in the low-FTA group, with a mean difference of 6.13° (*P* = .005), indicating a statistically significant difference between the 2 groups (Table 3).

**Table 3:** Comparison of femoral torsional angle (FTA) and tibial torsional angle (TTA) in the high-FTA and low-FTA groups: results from Student *t* test.

	High FTA group (mean ± SD)	Low-FTA group (mean ± SD)	*P* value
Femoral torsional angle	20.62° ± 5.46°	−12.74° ± 5.79°	<.001
Tibial torsional angle	34.3° ± 7.72°	28.17° ± 8.35°	.005

## 4. Discussion

### 4.1. Key results

This study proved the 3 hypothesis set in the study objectives as follows: the FTA of the excessive out-toeing squat group was lower than that of the general population; the TTA of the excessive out-toeing squat group was lower than that of the general population; and the TTA tended to decrease when FTA decreased.

Patients with difficulty in squatting often find vigorous physical activity challenging, and even if they can engage in such activities, they often experience hip pain. Therefore, these patients often visit outpatient clinics. Several patients exhibit an excessive out-toeing posture when squatting, except those with Achilles tightness. One of the causes of difficulty in squatting, as indicated in several studies, is GMC, which includes collagen disorders, genetic conditions, and iatrogenic injuries.^[19, 20]^ Degeneration, necrosis, and fibrosis of the gluteal muscles and fascia after various intramuscular injections are the main causes of hip joint motions, including flexion, adduction, and internal rotation.^[7, 21]^ However, as the diagnostic rate of GMC is low in South Korean patients, we focused on decreased femoral torsion as the main cause of excessive out-toeing squats. Moreover, we believe that the decrease in tibial torsion could also contribute to factors causing excessive out-toeing squats, as it could affect the toe and hip positions. To confirm this, we made the 3 assumptions mentioned in the introduction section and aimed to prove them in this study.

The well-recognized conditions restricting squatting include Achilles tightness, hip joint disorders, and patellofemoral disorders.^[22, 23]^ This study could shed light on lower-extremity torsional problems, especially decreased femoral anteversion, as a key contributor to the inability to squat. Moreover, we recommend that this be incorporated as a criterion in the pre-enlistment medical screening. By identifying such torsional problems in advance, individuals with occupations that require high-intensity training or athletes could prevent unnecessary injuries or hip pain during their training process.

### 4.2. Limitations

This study had a few limitations. First, the patient group data in this study were collected retrospectively from a single center by a single orthopedic surgeon, and the sample size was relatively small, with 25 patients and 50 hips. Due to the small sample size, there is a potential for measurement errors and statistical errors. However, to reduce these errors, interobserver and intraobserver reproducibility were verified. Furthermore, the difference in FTA is approximately 10 degrees, which suggests a significant difference, thereby reducing the possibility of statistical errors. If a larger number of patients had been recruited from multiple centers, more significant evidence to support the hypothesis could have been provided. Second, owing to the long data collection period and the inclusion of older imaging data, it was necessary to measure the FTA using axial cuts in CT instead of using 3D or oblique views. Using 3D or oblique views for measurements can more accurately reflect the actual FTA. In a cadaveric study by Fuller et al, the accuracy of 5 different methods for measuring FTA were compared. The method that showed the closest match to the CT 3D model involved measuring the FTA using an oblique view of the femoral neck. However, the same study also found that the axial view method was sufficiently accurate and demonstrated an excellent correlation with a 3D model.^[9]^ Therefore, our measurement method is considered appropriate for demonstrating this hypothesis. Lastly, because of the retrospective nature of the data collection, we were unable to establish a specific angle as the criterion for the out-toeing position in the patient group. During patient examinations, we defined excessive out-toeing squats as instances where patients exhibited excessive out-toeing positions while squatting compared to their usual walking foot position. In the squat assessment, the precise out-toeing angle was not measured, preventing the establishment of a correlation with FTA decrease. Thus, it was only confirmed that a decrease in FTA could be a cause of excessive out-toeing squat or inability to squat, without establishing a direct correlation. It is important to note that all patients in our study met this criterion. We believe that our study results effectively demonstrated FTA and TTA as the contributing factors to excessive out-toeing squats.

### 4.3. FTA differences between the 2 groups

To prove the first assumption that “the FTA of the excessive out-toeing squat group would be lower than that of the general population,” we compared the FTA of the patient group with that of the control group. The mean FTA of the patient group was 0.34, whereas that of the control group was 10.14°, indicating a statistically significant difference of 9.8°. Chiang et al^[2]^ were the first to propose that aberrant femoral torsion is the cause of excessive out-toeing squats. They referred to this squatting position as frog-leg squatting. The study was conducted on 67 patients who underwent MRI for suspected GMC, and 8 showed diminished femoral anteversion, whereas 11 showed femoral retroversion. Thus, they revealed that aberrant femoral torsion was the cause of frog-leg squatting in 30% of the patients. However, the authors only presented the proportion of patients with lower FTA values based on the average value for patients of the same age, as reported in previous studies,^[10]^ which did not reflect differences in average values due to race and other factors. In this study, we compared the measurements of the patient group with those of a control group randomly selected from the same race in the general population and demonstrated a significant difference, providing more solid evidence.

### 4.4. TTA differences between the 2 groups

To prove the second assumption, “the TTA of the excessive out-toeing squat group would be lower than that of the general population,” we compared the TTA of the patient group with that of the control group. The mean TTA of the patient group was 27.95°, whereas that of the control group was 32.67°, indicating a statistically significant difference of 4.72°. Hence, despite the small difference relative to FTA, TTA was verified to be lower in the excessive out-toeing squat group than in the general population.

### 4.5. Relationship between the FTA and TTA

To prove the third assumption, “if the FTA decreased, the TTA would also decrease,” we conducted a Pearson correlation analysis, but no significant correlation was observed. However, as mentioned earlier, we believe this may be due to the narrow measurement range and small sample size; hence, we proceeded with further analysis. We selected 30 individuals with the highest FTA values from the patient and control groups and categorized them as the high-FTA group. Similarly, we selected 30 individuals with the lowest FTA and categorized them as the low-FTA group. We then measured the TTA in each group to examine the potential relationship between FTA and TTA. The mean TTA of the high-FTA group was 34.3°, whereas that of the low-FTA group was 28.17°, a difference of 6.13° (*P* = .005), indicating statistical significance. Although this is not conclusive evidence of a direct association between FTA and TTA, it does suggest a tendency for TTA to decrease when FTA decreases.

In summary, we demonstrated that a decrease in the FTA was a significant contributing factor to excessive out-toeing squats. Although the patient group showed a TTA decrease, the difference from the general population was smaller than that of the FTA. Furthermore, we observed that a decrease in TTA accompanied a decrease in FTA. Considering this, it can be inferred that the decrease in TTA observed in patients with excessive out-toeing squats is associated with a decrease in FTA. This study is significant because it is the first to analyze decreased TTA in patients with excessive out-toeing squats and confirms its association with decreased FTA.

### 4.6. Clinical analysis of the relationship between femoral anteversion and the inability to squat

Considering the reasons behind the decrease in the FTA causing excessive out-toeing squats, we speculate that this is likely due to intra-articular and extra-articular hip impingement. To achieve normal anteversion of the proximal femur, which prevents impingement and enables hip flexion during squatting, it is necessary to adopt an out-toeing position. Intra-articular impingement refers to femoroacetabular impingement (FAI), whereas extra-articular impingement refers to impingement caused by insufficient space for soft tissues. Satpathy et al^[24]^ reported that a decrease in femoral torsion generally leads to an increased incidence of pincer-type FAI. However, in clinical practice, repetitive impingement is believed to lead to the development of a bump, resulting in the concomitant occurrence of cam-type FAI. Therefore, mixed-type impingements are often observed in these patients.^[25]^ Although this was a slightly different study, reports indicate that surgical outcomes for FAI are unfavorable in cases of femoral retroversion.^[22, 26]^ This also serves as supporting evidence for the association between decreased femoral torsion and FAI. Lerch et al^[27]^ published an interesting study in which they conducted a 3D-CT-based impingement simulation analysis. They reported that one-third of samples with decreased femoral torsion exhibited the coexistence of intra-articular and extra-articular sub-spine FAI. They proposed that extra-articular sub-spine FAI can be understood in a context similar to that of the extra-articular hip impingement mentioned earlier, which refers to the restriction of hip flexion due to insufficient space for soft tissues.

### 4.7. Application in therapeutic settings and real-world scenarios

First, the most crucial point is that the identification of decreased FTA as the cause of inability to squat can lead to a reduction in the number of patients requiring treatment before their condition worsens. In countries with compulsory military service, including decreased FTA as a criterion for military enlistment should be considered. If individuals without the ability to squat are enlisted into the military, they might be prone to injuries or chronic pain during training. Furthermore, it could diminish the overall military capability. In this regard, this study holds significant importance as it can provide the basis for preventing such issues, ensuring the well-being of individuals and the overall military effectiveness.

Since decreased FTA leading to the inability to squat may not significantly affect daily life, surgical intervention for this issue is deemed unnecessary. However, it is crucial for patients to be aware that such limited functional capacity could pose challenges in specific situations. In modern society, as the quality of life improves, individuals are exposed to various forms of exercise for maintaining their health. In such cases, if patients are aware of their limitations and avoid specific activities accordingly, unnecessary pain and associated treatment costs can be minimized. In future research, it will be essential to further investigate the frequency of patients who present with an inability to squat due to lower extremity torsional problems. While no specific causes seem evident for such issues, if any exist, they should be identified and explored with the anticipation that they might be treatable. Subsequent studies focusing on lower-extremity torsional problems are hoped to shed light on these aspects, potentially reducing unnecessary patient discomfort and medical costs.

Despite limitations, this study demonstrated that a decreased FTA is a cause of excessive out-toeing squats. By comparing this with the general population of the same race, we provided clearer evidence of FTA reduction. Patients with excessive out-toeing squats showed a tendency toward decreased TTA values. However, it can be inferred that this is not a significant independent cause of excessive out-toeing squats but rather a concomitant effect when the FTA decreases. The restriction of the squat position presents significant limitations in high-level physical activity, making it an important concern for athletes, soldiers, and other professionals. Further research is required to elucidate the underlying causes of this issue.

## 5. Conclusion

Patients with excessive out-toeing squat showed lower FTA and TTA values than the general population. Both showed a statistically significant difference, with the difference in FTA being more pronounced. Furthermore, although a correlation between FTA and TTA was not established through Pearson correlation analysis, a tendency was observed where a decrease in FTA was associated with a decrease in TTA. Based on these results, decreased FTA was demonstrated to be one of the major causes of excessive out-toeing squats. Future research using 3D-CT scans on a larger cohort of patients will be necessary to accurately determine the extent of FTA decrease that leads to an inability to squat.

## Acknowledgments

English language editing fee of this research was supported by the research project registered with the Catholic Industry-Academic Cooperation Foundation in 2023 (No. 5-2023-D0797-00001). We thank Editage (www.editage.co.kr) for English language editing.

## Author contributions

**Conceptualization:** Dong Hwan Lee, Seok Jung Kim.

**Data curation:** Dong Hwan Lee, Yun Hwan Kim, Jaeyoon Baek.

**Formal analysis:** Dong Hwan Lee, Yun Hwan Kim.

**Investigation:** Dong Hwan Lee, Seok Jung Kim.

**Methodology:** Dong Hwan Lee, Seon Ae Kim, Seok Jung Kim.

**Visualization:** Dong Hwan Lee, Jaeyoon Baek.

**Writing – original draft:** Dong Hwan Lee.

**Validation:** Seon Ae Kim, Seok Jung Kim.

**Supervision:** Seok Jung Kim.

**Writing – review & editing:** Seok Jung Kim.
